# Exosomes From Astrocyte Processes: Signaling to Neurons

**DOI:** 10.3389/fphar.2019.01452

**Published:** 2019-12-02

**Authors:** Arianna Venturini, Mario Passalacqua, Simone Pelassa, Fabio Pastorino, Mariateresa Tedesco, Katia Cortese, Maria Cristina Gagliani, Giuseppina Leo, Guido Maura, Diego Guidolin, Luigi F. Agnati, Manuela Marcoli, Chiara Cervetto

**Affiliations:** ^1^ Section of Pharmacology and Toxicology, Department of Pharmacy, University of Genova, Genova, Italy; ^2^ Section of Biochemistry, Department of Experimental Medicine, and Italian Institute of Biostructures and Biosystems, University of Genova, Genova, Italy; ^3^ Laboratory of Experimental Therapies in Oncology, IRCCS Istituto G. Gaslini, Genova, Italy; ^4^ 3BrainAG, Wädenswil, Switzerland; ^5^ Department of Informatics, Bioengineering, Robotics and System Engineering DIBRIS, University of Genova, Genova, Italy; ^6^ Section of Anatomy, Department of Experimental Medicine, University of Genova, Genova, Italy; ^7^ Department of Biomedical, Metabolic and Neural Sciences, University of Modena and Reggio Emilia, Modena, Italy; ^8^ Department of Neurosciences, University of Padova, Padova, Italy; ^9^ Department of Neuroscience, Karolinska Institutet, Stockholm, Sweden; ^10^ Centre of Excellence for Biomedical Research CEBR, University of Genova, Genova, Italy

**Keywords:** adult astrocytes, astrocyte processes, cerebral cortex, ex-vivo, exosomes, extracellular vesicles, neuroglobin, neuron-astrocyte cocultures

## Abstract

It is widely recognized that extracellular vesicles subserve non-classical signal transmission in the central nervous system. Here we assess if the astrocyte processes, that are recognized to play crucial roles in intercellular communication at the synapses and in neuron-astrocyte networks, could convey messages through extracellular vesicles. Our findings indicate, for the first time that freshly isolated astrocyte processes prepared from adult rat cerebral cortex, can indeed participate to signal transmission in central nervous system by releasing exosomes that by volume transmission might target near or long-distance sites. It is noteworthy that the exosomes released from the astrocyte processes proved ability to selectively target neurons. The astrocyte-derived exosomes were proven positive for neuroglobin, a protein functioning as neuroprotectant against cell insult; the possibility that exosomes might transfer neuroglobin to neurons would add a mechanism to the potential astrocytic neuroprotectant activity. Notably, the exosomes released from the processes of astrocytes maintained markers, which prove their parental astrocytic origin. This potentially allows the assessment of the cellular origin of exosomes that might be recovered from body fluids.

## Introduction

The relevance of neuron-astrocyte network function in the intercellular communication in central nervous system (CNS) as well as in the vulnerability to neurodegenerative and neuropsychiatric diseases is widely accepted (see Halassa and Haydon, 2010; Sofroniew, 2015; Verkhratsky et al., 2016). In the neuron-astrocyte networks the perisynaptic astrocyte processes function as sensors of transmitters in the extracellular environment—acted upon by neurotransmitters and gliotransmitters through a volume transmission mode of communication (see for reviews Agnati and Fuxe, 2000; Vizi, 2000)—and modulate neural activity by clearing glutamate and by releasing gliotransmitters (see Verkhratsky et al., 2016; Cervetto et al., 2018 and references therein); they also regulate extracellular space volume and coverage of synapses (Xie et al., 2013). Indeed, they represent the astrocyte compartment specially devoted to bidirectional neuron-astrocyte communication in the complex interaction involving pre- and post-synaptic elements (the tripartite synapse; Araque et al., 1999) with the extracellular matrix (the tetrapartite synapse; Thalhammer and Cingolani, 2014; Agnati et al., 2018) and to regulation of synapse plasticity.

An increasing amount of evidence indicates that extracellular vesicles (EVs) operate as carriers of signals in CNS. Intercellular communication through EVs is generally accepted as a mode of non-synaptic communication in CNS—the roamer-type of volume transmission—contributing to the role of the extracellular space in the signaling diffusion and codification in the brain (Agnati et al., 2014). Exosomes—EVs of about 30–100 nm diameter, released into the extracellular space upon fusion of multivesicular bodies with the plasma membrane—are recognized to play multiple roles in both physiological and pathological conditions in CNS (Février and Raposo, 2004; Raposo and Stoorvogel, 2013 and references therein). Various CNS cell types, including neurons, microglia and oligodendroglia, can release exosomes; while cultured astrocytes have been reported to secrete exosomes (see Taylor et al., 2007; Guescini et al., 2010; Wang et al., 2011; Wang et al., 2012; Guitart et al., 2016; Willis et al., 2017; Hira et al., 2018; Pascua-Maestro et al., 2019; Pei et al., 2019; Xu et al., 2019; see also Verkhratsky et al., 2016 and Lafourcade et al., 2016), less is known on the ability of astrocytes to release exosomes in neuron-astrocyte networks.

Here we investigate on the possibility that the processes of astrocytes might convey messages in non-classical mode through EVs. We report for the first time that astrocytic processes freshly prepared from adult rat cerebral cortex and originating from astrocytes that have matured in astrocyte-neuron networks, are provided with structures resembling multivesicular bodies and can release vesicles, which exhibit the features of exosomes. Therefore, although their subcellular origin cannot be directly demonstrated, the vesicles can be considered *bona-fide* exosomes (from now on, “exosomes”). Moreover, we report that the exosomes can transport neuroglobin (NGB). NGB, a protein produced mainly in neurons within the CNS but also detected in astrocytes, and exhibiting anti-oxidant, anti-apoptotic, and anti-inflammatory effects, might function as a neuroprotectant against hypoxic/ischemic insult, β-amyloid, or H_2_O_2_ toxicity (see Guidolin et al., 2014; Guidolin et al., 2016; Van Acker et al., 2019 and references therein). Noteworthy, the exosomes released from the astrocyte processes were able to selectively target neurons. The finding that astrocytic processes express and release NGB might contribute additional mechanisms to the astrocyte neuroprotective potential.

## Materials and Methods

### Chemicals and Reagents

Percoll, bovine serum albumin (BSA), poly-L-ornithine, PKH67 fluorescent cell linker kit (catalog number PKH67GL MIDI67), and all the salts were from Sigma-Aldrich St. Louis, MO USA. The primary or secondary antibodies were from Sigma-Aldrich [mouse anti-synaptophysin, catalog number: S5768; rabbit anti-glial fibrillary protein (GFAP), catalog number: G9269; mouse anti-GFAP (clone G-A-5), catalog number: G3893; mouse anti-ezrin, catalog number: E8897; mouse anti-β-actin, catalog number: A2228; rabbit anti-β III tubulin, catalog number: SAB4500088], from Synaptic Systems, Goettingen, Germany [rabbit anti-microtubule-associated protein 2 (MAP2), catalog number: 188 003], from Merck Millipore Corporation, Darmstadt, Germany [mouse anti-oligodendrocyte (RIP), catalog number: MAB1580; mouse anti-integrin-αM (clone OX-42), catalog number: CBL 1512], from Santa Cruz Biotechnology Inc, Dallas, TX USA [rabbit anti-NGB (clone FL-151); catalog number: sc-30144] or from Thermo-Fisher Scientific Inc, Waltham, MA USA [mouse anti-Alix (clone 3A9), catalog number: MA1-83977; mouse anti-Tsg101 (clone 4A10), catalog number: MA1-23296; Alexa-Fluor 488 or 633 conjugated goat anti-rabbit or anti-mouse secondary antibodies]. The horseradish peroxidase-linked anti-rabbit or anti-mouse secondary antibodies were from Cell Signaling Technology Inc, Danvers, MA USA. Prolong Gold Antifade Mountant were from Molecular Probes, Eugene, OR USA; the microporous filters and the polyvinylidene difluoride membrane were bought from Merck Millipore Corporation, Darmstadt, Germany. The mini gel used for western blot were from Bio-Rad Laboratories, Hercules, CA USA; ECL-PLUS kit was from GE Healthcare, Milano, Italy; Neurobasal, DMEM, B27, Glutamax, and Pen-Strepto were from Gibco by Thermo-Fischer Scientific Inc.

### Animals

Adult male rats (200–250 g, Sprague–Dawley) were housed at constant temperature (22 ± 1°C) and relative humidity (50%) under a regular light-dark schedule (lights on 7 AM–7 PM). Food and water were freely available. To prepare primary neuronal cultures Sprague–Dawley rat embryos at the day 18 of gestation (E18) were used. The pregnant dams were anesthetized and the embryos were extracted by caesarian section.

Animal care and experimental procedures complied with the European Communities Parliament and Council Directive of 22 September 2010 (2010/63/EU) and with the Italian D.L. n. 26/2014, and were approved by the Italian Ministry of Health (protocol number 26768 of November 2012 and protocol number 75F11.N.6JI of August 2018), in accordance with Decreto Ministeriale 116/1992. All efforts were made to minimize the number of animals used and their suffering, and no *in vivo* technique was used.

### Preparation of Purified Astrocytic Processes

Purified astrocyte processes (gliosomes) were prepared from the cerebral cortex of adult male rats. Briefly, after decapitation, the tissue was rapidly removed and placed in ice-cold medium. Purified gliosomes were prepared by a discontinuous Percoll gradient according to Nakamura (Nakamura et al., 1993) as previously reported (see Cervetto et al., 2018). Briefly, rat cerebral cortices were homogenized in 10 volumes of 10 mM Tris/HCl pH 7.4 with 0.32 M sucrose, using a glass-Teflon tissue grinder (clearance 0.25 mm). The homogenate was centrifuged (5 min at 4°C and 1,000 g) to remove nuclei and debris and the supernatant stratified on a discontinuous Percoll gradient (2, 6, 10, and 20% (v/v) in Tris-buffered sucrose) and centrifuged for 5 min at 4°C and 33,000 g. The layer between 2% and 6% (v/v) Percoll (gliosomal fraction; purified astrocyte processes) was collected and washed by centrifugation. For release experiments, purified astrocyte processes were suspended in standard HEPES medium (mM: NaCl 128, KCl 2.4, MgSO_4_ 1.2, KH_2_PO_4_ 1.2, CaCl_2_ 1.0, and HEPES 10 with glucose 10, pH 7.4). Protein determinations were carried out using serum bovine albumin as the standard (see Cervetto et al., 2018).

### Confocal Microscopy on Gliosomes and Synaptosomes

Immunofluorescence confocal microscopy on gliosomes and synaptosomes was performed according to sequential staining methods (see Cervetto et al., 2016; Cervetto et al., 2018). Briefly, gliosomes and synaptosomes were fixed and permeabilized in 2% paraformaldehyde (PFA)/0,1% Triton X-100 in phosphate buffer solution (PBS) pH 7.4 and then incubated in the diluted primary antibodies in 3% BSA in PBS (over-night, 4°C). The following primary antibodies were used: mouse anti-synaptophysin (1:1,000), rabbit anti-GFAP (1:1,000); mouse anti-RIP (1:10,000), and mouse anti-integrin-αM (1:25). After washing with PBS the preparations were incubated with the appropriate Alexa-Fluor 488 or 633 conjugated secondary antibodies (1:1,000). Gliosomes and synaptosomes were then smeared onto coverslips with anti-fade mounting medium (ProLong Gold). Images were collected by means of a three-channel TCS SP2 laser-scanning confocal microscope (Leica Wetzlar, Germany) using a plan apochromatic oil immersion objective 60×/numeric aperture 1.43. The ImageJ software (Wayne Rasband, National Institutes of Health, Bethesda, MD, USA) was used to count positive particles using 3D-counter object analyzed application (Threshold = 50 in all the fields—Fiji ImageJ). The percentage of GFAP, synaptophysin, RIP, and integrin-αM positive particles was estimated in three to five non-overlapping fields from three different preparations of gliosomes and synaptosomes, and are expressed as mean ± SEM.

### Release Experiments and Extracellular Vesicle Isolation and Characterization

We collected the EVs released from the astrocyte processes essentially by applying the method used to collect the gliotransmitters released from isolated perfused astrocyte processes (gliosomes) or the neurotransmitters released from isolated perfused nerve terminals (synaptosomes). Briefly, gliosomes were stratified on microporous filters (MF-Millipore™, Thickness: 180µm; Pore size: 0.65µm; Merck-Millipore) at the bottom of parallel perfusion chambers at 37°C and continuously perfused (0.5 ml/min) with a standard medium as described previously (Cervetto et al., 2017; Cervetto et al., 2018). After 5-min perfusion, perfusate fractions were collected in a 10-min sample. The perfusate was pelleted by ultracentrifugation at 110,000 g for 90 min (Guescini et al., 2010) and the EVs were resuspended i) in PBS to perform nanosight analysis by using a dynamic light scattering; ii) in loading buffer for performing western blot analysis; iii) in diluent C, according to PKH67 kit technical instructions, to be labeled with the exosome dye PKH67 for assessing their ability to target cells.

### Dynamic Light Scattering

To measure the size of the EVs released from astrocyte processes we performed the nanosight analysis on ultracentrifugation pellet resuspended in PBS using the Zetasizer Nano ZS90 particle sizer at a 90° fixed angle (Malvern Instruments, Worcestershire, United Kingdom), as previously described (Marimpietri et al., 2013). Nanosphere™ size standards with a mean diameter of 57 ± 4 nm (Thermo Scientific) were used for particle sizer calibration. The analysis was replicated on three different samples.

### Western Blot

The western blot analysis was performed both on gliosomes, synaptosomes, and EVs. Proteins were denatured in Laemmli sample buffer and then subjected to a SDS-polyacrylamide gel electrophoresis (13% or 4–20% gradient mini gel) 200 V for 50 min (gliosomes: 5–20 µg/lane; synaptosomes: 10µg/lane; EVs: estimated amount of proteins: 2.53–6.32 µg/lane; Mini-Protean TGX Gel, Bio-Rad Laboratories), followed by electroblotting (100 V for 50 min) on polyvinylidene difluoride membrane (Immobilon-P PVDF; Millipore Corporation). The blot has been cut probing different regions of the same blot with multiple antibodies. Immunodetection was performed using the following primary antibodies: mouse anti-Alix (1:1,000); mouse anti-Tsg101 (1:800); rabbit anti-NGB (1:300); mouse anti-GFAP (1:1,000); mouse anti-ezrin (1:500); rabbit anti-MAP-2 (1:1,000); and rabbit anti-β III tubulin (1:1,000). Primary antibodies were incubated over-night at 4°C followed by washing and the application of horseradish peroxidase-linked anti-rabbit or anti-mouse (Cell Signaling Technology) secondary antibodies, incubated for 1 h at room temperature. Western blots were developed with the ECL-PLUS kit (GE Healthcare), according to the manufacturer's instructions. Band detection and densitometry were performed using the Chemi-Doc System and the quantity one software package (Bio-Rad Laboratories). The membranes were stripped using Re-blot plus solution (Merck-Millipore Corporation) and re-probed with mouse anti-β-actin (1:10,000) also to estimate the amounts of proteins in exosomes. Developed films were analyzed using specific software (GelDoc; Bio-Rad Laboratories).

### Electron Microscopy

Ultrastructural analysis of gliosomes and exosomes was performed by negative staining method. Briefly, 5µl drops of gliosomes or purified exosomes were placed onto formvar and carbon-coated copper grids and adsorbed for 20 min at room temperature. The excess of buffer was removed by using a filter paper. Then, grids were fixed in 2% PFA in PBS pH 7.2 for 5 min and washed out three times on large drops of distilled water. Grids were then incubated for 5 min at room temperature with 1% aqueous solution of uranyl acetate. Contrast enhancement was obtained by further incubating the grids with a mixture of 1% uranyl acetate and 1% methylcellulose for 5 min. After drying, grids with gliosomes or exosomes were immediately observed with a CM10 electron microscope (Philips, Eindhoven, The Netherlands). Digital images were taken with a Megaview II camera.

### Labeling of Exosomes

For immunofluorescence analysis, the exosome pellet was resuspended in diluent C and stained with the dye PKH67 according to the producer's technical bulletin (Fitzner et al., 2011). The dye was gently pipetted with the sample, and after 5 min at room temperature, the staining reaction was stopped bringing the volume up to 35 ml with 10% BSA in PBS. Exosomes were pelleted by ultracentrifugation (110,000 g for 90 min) and resuspended in 150 µl of Neurobasal. In parallel as control condition, we prepared the control samples with equal volumes of PBS plus the same amount of diluent C, PKH67 dye, 10% BSA and PBS, and by omitting the exosomes, to exclude any non-specific labeling of cells by micelles of the aliphatic dye or by the excess of dye.

### Neuron-Astrocyte Co-Cultures

Primary cortical cells were derived from Sprague–Dawley rat embryonic day 18 (E18). Culture preparation was performed as previously described (Chiappalone et al., 2006). Briefly, E18 timed pregnant Sprague–Dawley rat was euthanized by CO_2_ and cervically dislocated in accordance with institutionally approved animal care. Embryos were dissected and cortices isolated in Hank's buffer solution without Ca^2+^ and Mg^2+^. All tissue was collected and maintained in ice-cold buffer solution and, to obtain a single-cell suspension, cerebral cortices were enzymatically digested at 37°C with warm TrypLe Express for 15–18 min in a water bath. The digestion was stopped by adding medium (Neurobasal or DMEM) complemented with 10% FCS (fetal calf serum) for 3 min, after this interval the medium was carefully removed and the cortices, transferred in Neurobasal/B27 (supplemented with Glutamax and Pen-Strepto), were mechanically triturated with a sterile fire-polished Pasteur pipette. Single-cell suspension was well mixed, counted, and diluted. Finally, cells were plated on poly-L-ornithine (100 µg/ml) coated coverslips inserted into multiwells plates at the density around 5.0x 10^4^cell/cm^2^. The primary cultures were kept at 37°C in humidified atmosphere of 5% CO_2_ in air. The culture medium was changed weekly, until the uptake experiments at 21 DIV, at the end of the 3 weeks of development of the *in vitro* culture the percentage composition of the cell population was distributed with 70 ± 15% of neurons and 30 ± 15% of glial cell (the percentage of neurons and astrocytes were estimated in three to five non-overlapping fields from three different cultures, and are expressed as mean ± SEM), consistent with previous findings (Chiappalone et al., 2006). Immunofluorescence assays were performed using rabbit anti-MAP-2 (1:500), mouse anti-GFAP (1:1,000), and DAPI dye. See a representative image acquired by epifluorescence microscopy in **Figure 3A**.

### Cellular Uptake of Exosomes

Exosomes, made fluorescent with the PKH67 lipophilic dye, were dispersed (16 µl) in the same culture medium where the coverslips were immersed with the neuronal networks and incubated for 1 h at 37°C in humidified atmosphere of 5% CO_2_ in air.

In parallel, as a negative control, the same volume (16 µl) of control sample was loaded onto other coverslips from the same neuronal preparation, and left to incubate for 1 h under the same conditions as above. After the time interval had elapsed, both the coverslips with the exosomes and those with control sample were washed repeatedly to remove the excess.

To evaluate the uptake capabilities of the exosomes, the experiments were repeated on three different neuronal preparations, developed *in vitro* for 3 weeks, and the biological sample was subjected to specific marking to confirm its neuronal/glial morphology and exosomes uptake. Briefly, the cells were fixed in 4% PFA, blocked with 3% BSA, and incubated primarily with rabbit or mouse primary antibodies (over-night at 4°C in humid chamber) and then with Alexa Fluor 546 donkey anti-mouse and 633 goat anti-rabbit (1h at room temperature). The following primary antibodies were used: rabbit anti-β III tubulin (1:500) or rabbit anti-MAP2 (1:500), or rabbit or mouse anti-GFAP (1:1,000). The excessive antibodies were washed by PBS. The glass coverslips were mounted with antifade mounting medium and observed using confocal microscopy (see above).

## Results

### Gliosomes Obtained From Adult Rat Cerebral Cortex Are a Purified Preparation of Cerebrocortical Astrocyte Processes

**Figure 1:**
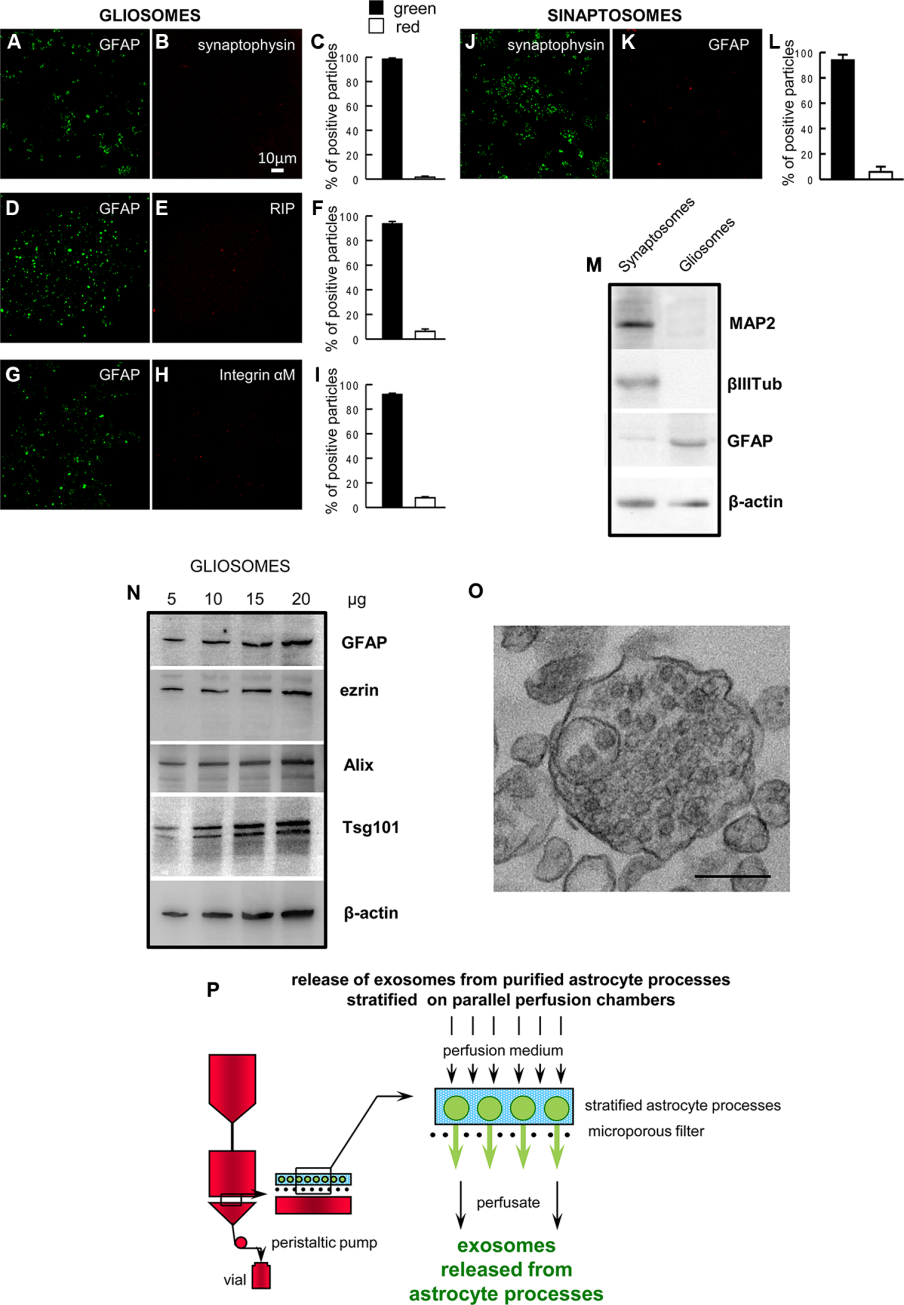
Astrocyte processes obtained from adult rat cerebral cortex. Negligible contamination of gliosomes, positive for the specific glial marker GFAP **(A**, **D**, **G)**, by subcellular no-astrocytic particles. Immunofluorescent assay for synaptophysin **(B)**, RIP **(E)** or integrin-αM **(H)** markers for nerve terminals, microglia, and oligodendrocytes, respectively. As a positive control, the immunofluorescent assay for synaptophysin **(J)** was performed on cerebral cortical synaptosomes scarcely contaminated by subcellular GFAP-positive particles **(K)**. Bars **(C**, **F**, **I**, **L)** represent the percent of positive particles (% ± SEM of positive particles counted in three to five no-overlapping fields from n = 3 different preparations): GFAP (C, F, I, solid bars; L, empty bar), synaptophysin (C empty bar; L solid bar; L, empty bar), or RIP or integrin-αM (F or I, respectively; empty bar). Scale bars are indicated in the figures. Western blot analysis of gliosomes and synaptosomes **(M**, **N)**. The absence of cross-contamination of the astrocyte processes and nerve terminals is shown **(M)**: MAP2, β III tubulin, and GFAP proteins were used as selective markers for the synaptosome or gliosome preparations. Presence of the astrocytic markers GFAP and ezrin, and of the exosome markers Alix and Tsg101 in the gliosomes **(N)**. Electron microscopy image of a cortical astrocyte processes. A single gliosome is shown containing vesicles scattered in the cytoplasm and a multivesicular body **(O)**. Scale bars: 200 nm. Schematic of a perfusion unit of the apparatus allowing recovery of extracellular vesicles (exosomes) released from the processes during perfusion **(P)**. For other experimental details, see *Materials and Methods*.

At confocal microscopy, the astrocyte processes appeared labeled with the anti-GFAP antibody (a marker identifying astrocytes), and were negative for synaptophysin, integrin-αM, and RIP (markers for the nerve terminals, microglia, or oligodendrocytes, respectively. **Figures 1A**–**I**). As a control, we also show that the nerve terminals (synaptosomes) prepared from rat cerebral cortex were positive for synaptophysin and negative for GFAP (**Figures 1J**–**L**). The Western blot analysis showed the absence of contamination by neural specific proteins (MAP2 and β III tubulin) in the gliosome preparation, and the negligible contamination of the cerebral cortical synaptosomes prepared in parallel (**Figure 1M**). The findings indicate that gliosomes are a purified preparation of processes of cerebrocortical astrocytes, negligibly contaminated by neuronal, microglial, or oligodendroglial particles. The processes were also analyzed by Western blot and were found to express the astrocytic markers GFAP and ezrin, the exosome endosomal-lysosomal sorting proteins Alix and Tsg101, and NGB (**Figure 1N**). The electron microscopy analysis on gliosomes revealed the presence of multivesicular bodies and scattered vesicles inside the astrocyte processes (**Figure 1O**).

### Purified Astrocytic Processes Release Extracellular Vesicles Exhibiting the Characteristics of Exosomes

**Figure 2:**
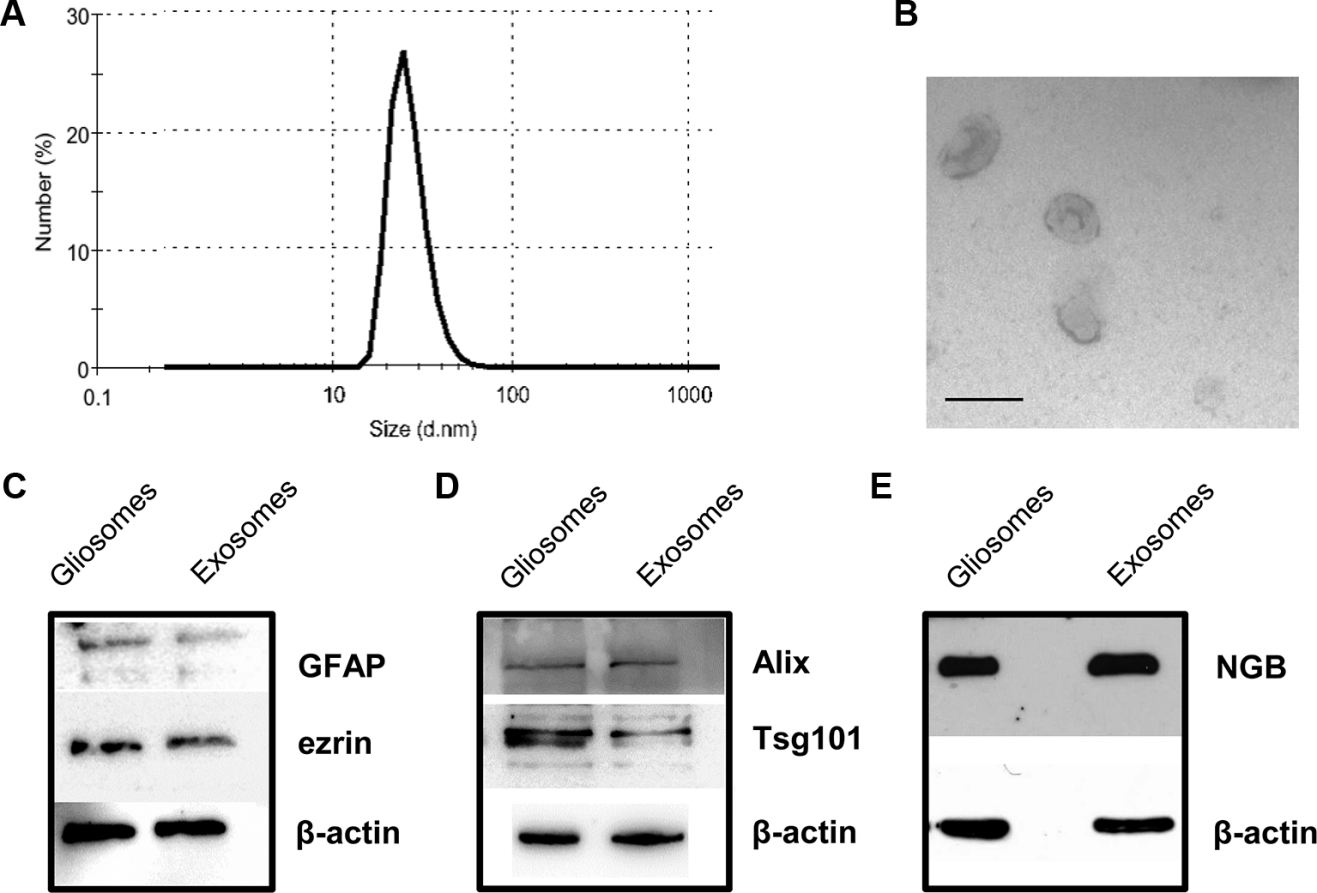
Characterization of rat cerebrocortical astrocyte processes-released exosomes. Size distribution of exosomes released from the astrocyte processes, as assessed by the zetasizer nano ZS90 particle sizer. Curve shows a representative tracing (from three samples obtained from three different experiments with similar results) **(A)**. Electron microscopy images of vesicles released from astrocyte processes. Note the cup shape appearance and size, consistent with previously reported exosome electron microscopy images characteristics **(B**; scale bar: 100 nm). Presence of astrocytic markers, exosomal markers, and of NGB **(C**–**E)**. Western blot for the astrocytic markers GFAP and ezrin in gliosome preparation and in exosomes released from gliosomes **(C)**. Western blot for the exosomal markers Alix and Tsg101 in gliosome preparation and in gliosome-released exosomes **(D)**. Western blot for NGB in gliosome preparation and in gliosome-released exosomes **(E)**. For other experimental details, see *Materials and Methods*.

The EVs released and recovered in the perfusate from cerebrocortical astrocyte processes (see **Figure 1P** for a scheme of perfusion unit) were firstly analyzed using nanosight dynamic light-scattering analysis and electron microscopy imaging, and subsequently for the presence of the exosome specific protein markers Alix and Tsg101. At dynamic light-scattering analysis, the EVs showed a bell-shaped size distribution profile, peaking at mode 60 nm (range 50–75) (see in **Figure 2A** the tracing of a representative experiment from three different experiments with similar results). The observed size is consistent with the theoretical size of exosomes and previous observations (Skog et al., 2008). The typical cup shape appearance at the ultrastructural level and their size (electron microscopy images, **Figure 2B**) are consistent with previously reported exosome electron microscopy images (see Raposo and Stoorvogel, 2013). The EVs were verified for the presence of astrocytic markers, namely for GFAP and ezrin. Using western blot analysis, we obtained signaling for both GFAP and ezrin in the EVs recovered from the perfusion collected samples (**Figure 2C**), demonstrating the astrocytic source of the particles collected.

Both the exosome specific protein markers Alix and Tsg101 were present in the vesicles (**Figure 2D**), confirming that the EVs recovered in the perfusate from the processes exhibit the features of exosomes.

Gliosomes and exosomes were also labeled with anti-NGB antibody (**Figure 2E**), indicating that exosomes carry NGB protein.

### The Released Exosomes Selectively Target Neurons and Can Be Internalized by Neurons

**Figure 3:**
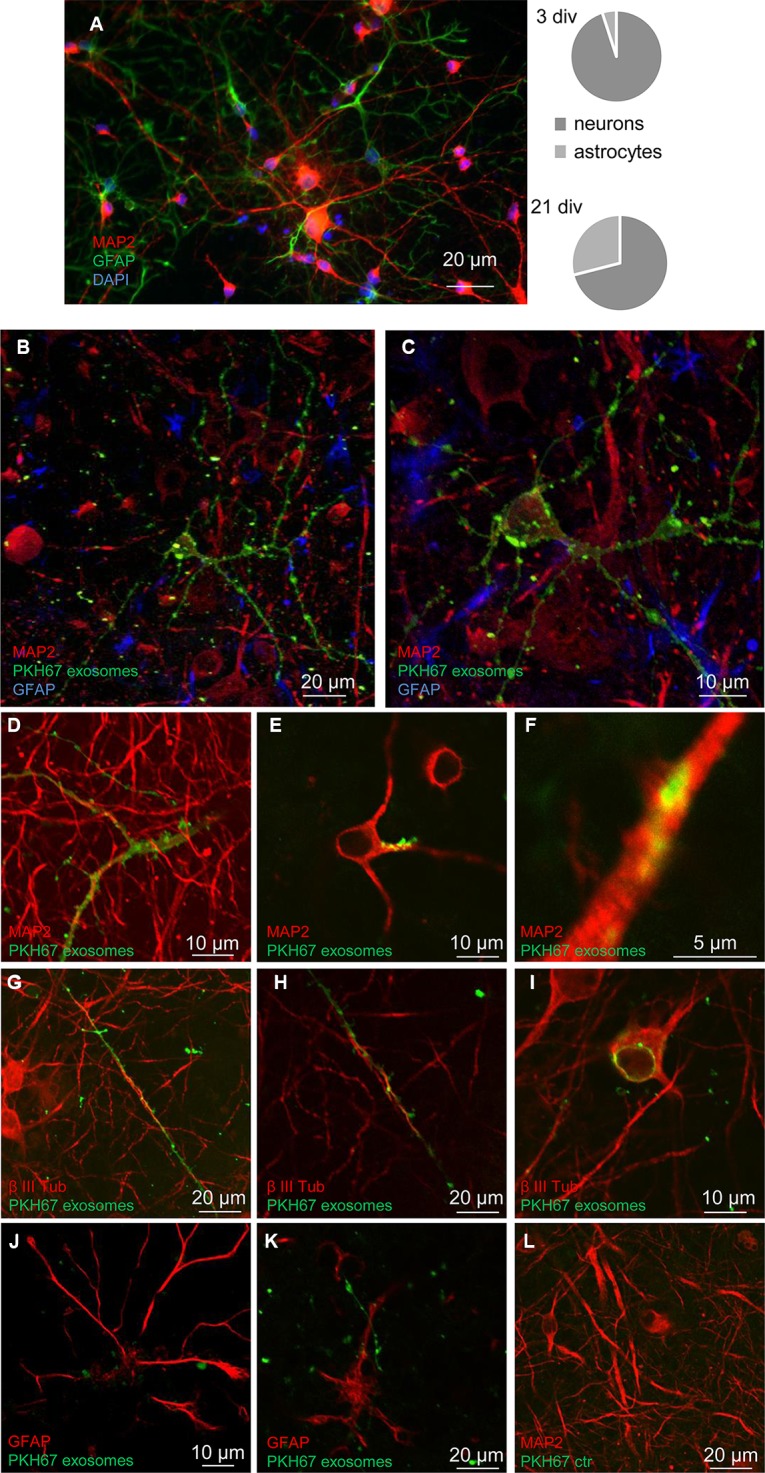
Neurons: targets for the exosomes. Confocal images showing exosomes targeting neurons when added to a neuron-astrocyte co-culture. Characteristics of the neuron-astrocyte co-culture; see coexistence of GFAP-positive (green) astrocytes and MAP2-positive (red) neurons in a representative epifluorescence microscope image at 21 div and their relative distribution at 3 and 21 div culture **(A)**. DAPI stained was used to marker cellular nucleus. Scale bar is indicated in the figure. See that exosomes (marked with PKH67, green) preferentially contact GFAP-negative cells, while GFAP-positive astrocytes are not targeted (blue in B, C, red in J, K). Exosomes selectively target cells positive for the neuronal markers MAP2 (in **B**, **C**, **D**–**F)** or β III tubulin (red in G-I). The images are the merge of a single z stack of the two channels **(D**–**F**, **H**–**L)** or representative maximum intensity projections of the acquired z stacks of the two or three channels **(B**–**C**; **G)**. See in **Video 1** the z-axis analysis related to the panel B. Note internalization of exosomes **(D**–**I)**: evidence for exosome presence inside the neuronal projections **(D**–**H)** and at the perinuclear region **(I)**. A control sample prepared in parallel by omitting the exosome excluded the non-specific labeling of cells **(L)**. Scale bars are indicated in the figures. For other experimental details, see *Materials and Methods*.

The exosomes released and recovered in the perfusate from cerebrocortical astrocyte processes were able to target cells in neuron-astrocyte co-cultures. Notably, in the co-cultures, only GFAP-negative cells were targeted by the exosomes, while GFAP-positive astrocytes were not. In particular, we found that exosomes targeted cells exhibiting the morphological features of neurons (**Figure 3**); labeling with the neuronal markers MAP-2 and β III tubulin confirmed the selective transfer of astrocyte-released exosomes to neurons (**Figures 3B**–**I**; **Video 1**). Notably, confocal microscopy confirmed the ability of exosomes to be internalized rather than being attached to the surface of the neuronal membrane (**Figures 3B**–**H**); we found evidence for exosome presence inside the neurons both at their projections (**Figures 3B**–**H**) and at the perinuclear region (**Figure 3I**). Interesting to note, exosome traveling to perinuclear region was already reported in PC12 cells as well as in human-induced pluripotent stem cells or in human neuroblastoma cell lines (Tian et al., 2010; Sardar Sinha et al., 2018).

## Discussion

Our main finding is represented by the fact that exosomes can be released from astrocyte processes and selectively target neurons; these exosomes might transfer NGB of astrocytic origin. The relevance and the novelty of these findings are to be considered in light of the following considerations:

-Although it was already demonstrated that exosomes can be released from cultured astrocytes (Guescini et al., 2010), astrocytes in culture can only marginally mimic the behavior of astrocytes in situ. We here report on processes of astrocytes acutely prepared from adult rat cerebral cortex, thus reflecting the behavior of astrocyte processes in mature cerebrocortical neuron-astrocyte networks. Notably, these processes were positive for ezrin, an astrocytic cytoskeletal protein selective marker of the perisynaptic astrocyte processes, required for the astrocyte processes structural plasticity (see Cervetto et al., 2018 and references therein). In the perfusate from the processes we collected particles that proved to be positive for the specific protein markers for exosomes, the endosomal-lysosomal sorting proteins Alix and Tsg101, indicating that exosomes can be released from the processes. In fact, electron microscopy imaging indicated the presence of multivesicular bodies in the processes, consistent with their ability to release exosomes. Astrocyte processes might therefore participate in a roamer-type of volume transmission through the release of exosomes. Thus, the processes are capable of contributing in multiple modes to the signal transmission in CNS, both receiving messages and sending messages of different type, and presumably with different half-life and targets, such as the gliotransmitters (e.g. glutamate, that can be rapidly taken up and/or activate non synaptic glutamate receptors) and signals that may be transferred through EVs. Notably, perisynaptic processes exhibit plasticity and can rapidly change their morphology, modifying the coverage of pre- and postsynaptic elements at the synapses (Reichenbach et al., 2010; Bernardinelli et al., 2014); plasticity of perisynaptic processes has been reported to result in dramatic changes of the interstitial space during sleep or pharmacological anesthesia (Xie et al., 2013). We can hypothesize that the conceivable consequent opening of the synapses might reduce the "privacy" of synaptic wiring transmission in favor of volume transmission, suggesting that astrocyte signaling through volume transmission might have different relevance and functions depending on physiological cycles and the state of synaptic coverage. This would contribute to the shift from a neurocentric to a neuro-astrocentric view of the brain functioning, as the perisynaptic astrocyte processes may be the source for both classical volume transmission through the release of gliotransmitters, and for roamer-type volume transmission through the release of exosomes. By this way, the astrocyte processes might be capable of inducing transient phenotype changes in the receiving cells (see Agnati et al., 2014 and references therein).

The exosomes released from the astrocyte processes were found to target neurons. It was already shown that exosomes from cultured astrocytes could contact *co-*cultured neurons to promote neurite outgrowth of neighboring neurons and/or neuronal survival (see Janas et al., 2016 and references therein; Frühbeis et al., 2012; Caruso Bavisotto et al., 2019) and could protect neurons against ischemic damage (see Hira et al., 2018; Pei et al., 2019; Xu et al., 2019). This is however to our knowledge, the first evidence indicating that *ex-vivo* astrocytes—in particular, astrocyte processes acutely prepared from adult astrocytes that have matured in neuron-astrocyte network—can selectively communicate to neurons through exosomes. In the framework of the complex bidirectional signaling coordinating the function of the neuron-astrocyte networks the evidence that the astrocyte processes can release exosomes to target neurons adds a further mode of astrocyte-to-neuron communication that might be of significant (and so far uncovered) importance in physiological as well as in pathological conditions. As a matter of fact, exosomes transfer from astrocyte might result in a transient phenotype change of the receiving neurons, e.g. by enrichment in neuroprotective factors (see below) or by expression of receptors (see exosomes carrying functionally competent neurotransmitter receptors to receiving cells; Guescini et al., 2012) making neurons transiently able to recognize and decode extracellular signals, with possible relevant neuropharmacological implications. The ability of astrocyte-derived exosomes to effectively transfer signals and functions to neurons is a crucial point worth to be investigated in the future. Also, it remains not understood why some neurons (and their projections) are preferentially targeted by exosomes; further investigation is required to understand the neurochemical characteristics or the attracting pathways of the neurons/neuronal projections to which exosomes preferentially bind.

-The exosomes were found to carry NGB. As a matter of fact, it was initially thought that NGB in mammals was expressed exclusively in neurons of the nervous system. NGB, however, was also observed in astrocytes and reactive astrocytes (see Della Valle et al., 2010 and references therein). In particular, it was hypothesized that NGB may be produced by astrocytes for secretion, possibly as a neuroprotective agent for neurons (Della Valle et al., 2010). Astrocytes, indeed, are recognized to play multiple roles in diverse pathological conditions in the brain, playing both neuroprotective and detrimental roles (see Verkhratsky et al., 2016) The possibility that astrocyte processes could release NGB through exosomes would allow them to send long-distance messages to cells, to transiently change their susceptibility to damage, and to participate in the beneficial effects of astrocytes in ischemic injury (see Verkhratsky et al., 2016 and references therein).

Actually, NGB can serve multiple crucial roles in cell defense and resistance to degeneration, and transferring NGB from astrocytes might contribute to protecting neurons. In this respect, it was reported that estradiol regulates NGB expression both in neurons and astrocytes through ERβ-mediated mechanisms and that this regulation of the expression of NGB may be part of the neuroprotective mechanisms activated by estradiol in astrocytes (see references in Guidolin et al., 2014; Guidolin et al., 2016). As a matter of fact, transferring signals through exosomes has been proposed to be involved in the participation of glial cells to neurodegeneration or neuroprotection (see Verkhratsky et al., 2016; Lafourcade et al., 2016); by supporting the ability of astrocyte-derived exosomes to target neurons, our findings indicate that astrocytes might participate to neuron neuroprotection by transferring NGB through this mode of astrocyte-neuron communication. Notably, the roles for exosomes in transferring protective signals to neurons were already suggested on the basis of data from cultured astrocytes (Taylor et al., 2007; Wang et al., 2011; Guitart et al., 2016; Hira et al., 2018; Pascua-Maestro et al., 2019; Pei et al., 2019; Xu et al., 2019).

-Exosomes, being released from a variety of cells, have been proposed as peripheral markers for diagnostic-prognostic purposes in various diseases. They have been also proposed as peripheral markers for CNS diseases; however, one of the problems in their reliability as markers, besides the correct classification of exosomes, is their origin (Raposo and Stoorvogel, 2013). It is to note that exosomes recovered in the blood and originating from astrocytes were reported to behave as marker for stress-induced disease (Gómez-Molina et al., 2019). Also, recently GFAP-positive exosomes originating from astrocytomas were found in the blood and were claimed to be of help to the glioma classification (Van Bodegraven et al., 2019). Furthermore, astrocyte-derived EVs were found in periphery in neuroinflammatory conditions or after brain focal radiation (Dickens et al., 2017; Willis et al., 2017; Cai et al., 2017). In addition to functioning as biomarkers of different pathological conditions, the astrocyte-derived exosomes in blood might also target peripheral organs in the brain-to-periphery signaling (Dickens et al., 2017; Cai et al., 2017; see also Gómez-Molina et al., 2019). Worthy of note, we here indicate that a subcellular region of astrocytes—the processes that are devoted to sending/receiving signals in the nervous system—might be primarily involved in signal communication through exosomes. Notably, the finding that astrocyte-derived exosomes are positive for astrocytic markers allows hypothesizing that analysis of these markers could help to understand the cellular origin of (parental cells originating the) exosomes that might be recovered in peripheral blood from healthy or diseased CNS.

## Conclusions

In conclusion, our findings for the first time indicate that the astrocyte processes acutely prepared from astrocytes matured in a neuron-astrocyte network in CNS might participate to signal transmission by releasing exosomes, which, in turn might target near or long-distance targets by volume transmission. The exosomes released by the processes proved to selectively target neurons, adding a new non-conventional mode of astrocyte-to-neuron signal transmission, with unexplored impact on integrative communication in the CNS and neuropharmacological implications. Indeed, releasing NGB-carrying exosomes might be a mode for astrocytes to operate as a signal to protect neighboring cells in neuron-astrocyte networks. Also, our findings could help to understand the parental cell origin of the exosomes that might be recovered from peripheral blood.

## Data Availability Statement

The datasets generated for this study are available on request to the corresponding author.

## Ethics Statement

The animal study was reviewed and approved by Organismo Preposto al Benessere Animale OPBA, University of Genova and Italian Ministry of Health: protocol number 26768 of November 2012 (tissue preparation from humanely sacrificed adult rats); protocol number 75F11.N.6JI of August 2018 (primary neuron-astrocyte cultures from E18rats), in accordance with Decreto Ministeriale 116/1992. No *in vivo* experiment was performed.

## Author Contributions

GM, DG, LA, MM, and CC initiated the project. MM and CC designed the experiments. AV, SP, and CC performed the animal experiments. AV, SP, GL, and CC performed isolation and mark of exosomes. AV and MP performed the Western blot. FP performed the nanosight dynamic light-scattering analysis. KC and MG performed ultrastructural analysis. MT and CC performed cell cultures and exosome uptake experiments. MP, MT, and CC performed cell imaging. AV, SP, GL, and CC analyzed the data. MP, FP, MT, GM, MM, and CC wrote the manuscript. DG and LA revised the manuscript. All authors read and approved the final manuscript.

## Funding

This work was supported by the University of Genova [Grant number 020301002054 to MM, Grant numbers D31J1100003005 and D31J1100161005 to CC]; and the University of Padova [Grant number 60A06-0481/14 to DG]. The funding sources had no involvement in study design; in the collection, analysis and interpretation of data; in the writing of the report; and in the decision to submit the article for publication.

## Conflict of Interest

Author MT was employed by company 3BrainAG, Wädenswil, Switzerland.

The remaining authors declare that the research was conducted in the absence of any commercial or financial relationships that could be construed as a potential conflict of interest.

## Abbreviations

CNS, central nervous system; EVs, extracellular vesicles; GFAP, glial fibrillary protein; NGB, neuroglobin; BSA, bovine serum albumin; MAP-2, microtubule-associated protein 2; PFA, paraformaldehyde; PBS, phosphate buffer solution; β III Tub, *β III* Tubulin

## Supplementary Material

Video 1 Neurons: targets for the exosomes. A z-axis analysis of astrocyte-derived exosomes marked with PKH67 (green) co-localization with MAP2 (red) in primary astrocyte neuron co-culture (scanning 0.14 μm z axis; 50 stacks). The merge images of each z field were imported as a sequence and then saved as a movie with three frames per second (ImageJ). GFAP (blue).
